# Carnauba Wax and Beeswax as Structuring Agents for Water-in-Oleogel Emulsions without Added Emulsifiers

**DOI:** 10.3390/foods12091850

**Published:** 2023-04-29

**Authors:** Ivana A. Penagos, Juan Sebastian Murillo Moreno, Koen Dewettinck, Filip Van Bockstaele

**Affiliations:** Vandemoortele Centre ‘Lipid Science and Technology’, Food Structure and Function Research Group (FSF), Ghent University, Coupure Links 653, 9000 Ghent, Belgium; ivana.penagos@ugent.be (I.A.P.);

**Keywords:** carnauba wax, beeswax, W/O emulsions, water-in-oleogel emulsions, Pickering emulsion, wax crystal

## Abstract

This research aims to explore the potential of waxes as ingredients in the formulation of food-grade water-in-oleogel emulsions without added emulsifiers. The effects of the wax type, wax concentration and water concentration were tested on systems containing exclusively water, sunflower oil, and wax. Beeswax and carnauba wax were used in the formulation of water-in-oleogel emulsions with 20%, 30% and 40% *w*/*w* of water. For the continuous phase, three different levels of wax were used, namely 50%, 100%, and 150% of the critical gelling concentration. More specifically, carnauba wax emulsions were prepared at 2.5%, 5.0% and 7.5% of wax, while concentrations of 0.75%, 1.5% and 2.25% of wax were utilized for the beeswax experiments. Samples were assessed over time regarding stability, rheology and microstructure (polarized light microscopy, cryo-scanning electron microscopy and confocal scanning laser microscopy). Our findings suggest that, if present in sufficient concentration, carnauba wax and beeswax can stabilize emulsions in the absence of additional added emulsifiers. The resulting systems were inherently different based on the wax used, as crystal morphology and droplet configurations are determined by wax type. The yield strain was dictated by the nature of the wax, while the complex modulus was mostly influenced by the wax concentration. To test the scaling-up potential, systems were crystallized in a pilot-scale scraped surface heat exchanger, resulting in notably smaller crystal sizes, reduced rigidity and a storage stability of over one year. These findings represent a starting point for the formulation of scalable water-in-oleogel emulsions without added emulsifiers.

## 1. Introduction

Emulsions are metastable systems that tend to separate over time. Low-molecular-weight surfactants and biopolymers are traditionally used to stabilize emulsions kinetically. Over the last decade, a novel approach to emulsion stability, which consists of using particles as colloid stabilizers, has been increasingly studied [1]. “Pickering stabilization” is the term commonly used to describe emulsions in which amphiphilic particulate materials are placed in the oil–water interface [2]. For W/O emulsions, natural waxes are a promising alternative, as they are mostly hydrophobic, food-grade and readily available. Altogether, this paper will try to assess whether natural waxes can stabilize water-in-oil (W/O) emulsions in the absence of added surfactants.

Natural waxes are complex mixtures of long-chain compounds, including hydrocarbons, free fatty alcohols, free fatty acids, and wax esters [3]. The exact composition differs between waxes of different sources (i.e., beeswax vs. carnauba wax). Consequently, their properties will differ (e.g., gelling capacity, melting behavior, etc.). For instance, carnauba wax (CRW) is mostly constituted by wax esters and free fatty alcohols. In contrast, the chemical composition of beeswax (BZW) is high in wax esters and hydrocarbons [3]. For more information on the compositional data of waxes, the reader is referred to Doan, et al. [4].

An increasingly studied benefit of waxes is their structuring potential. In fact, in recent years, a lot of attention has been paid to reformulating fat-rich products to reduce the total amount of saturated fatty acids (SFA) [5]. The main driver for this is the incidence of SFAs and the subsequent risk of non-communicable diseases, such as cardiovascular heart diseases [6]. Therefore, wax oleogels are often studied as potential replacers of saturated fats [3,5]. Ultimately, the intention is to develop foods that are low in saturated fatty acids and have no added emulsifiers, and that can maintain the desired quality characteristics historically associated with edible soft matter W/O emulsions.

Although substantial efforts have been made to study wax oleogelation and water-in-oleogel emulsions, most of the work has been performed in systems utilizing added emulsifiers [7,8,9,10,11,12,13,14,15]. Only a handful of studies have attempted formulations in the absence of added surfactants [16,17,18,19]. In 2009, Binks and Rocher tested the ability of wax microparticles to form W/O emulsions. Their research used carnauba wax (CRW) and reported that the instant cooling of a pre-emulsion (made of molten wax, oil, and water) is crucial in order to increase stability [16]. In 2013, a study by Patel et al. described a similar system in which no surfactant was needed to stabilize the oil and water interface. The emulsions combined rapeseed oil, shellac wax, and water, and remained stable for 18 weeks with a fixed droplet size distribution. Stability was attributed to a combination between the Pickering emulsion and network stabilization [18]. In 2016, Szumała and Luty reported that it was possible to create stable W/O emulsions using wax particles with no added surfactants. CRW, paraffin wax, and ceresin wax systems were studied at different ratios of wax, water, and paraffin oil. Stable emulsions (<90 days) were obtained at appropriate ratios of wax, oil and water. Their study suggests that CRW displayed surface-active properties as the wax was mostly located at the droplet interface [19]. Finally, Gao et al. (2021) recently reported that beeswax (BZW) could stabilize emulsions without added surfactants for over 60 days of storage. The stability was attributed to the beeswax crystals distributed in the continuous phase, rather than a Pickering stabilization effect. The authors also reported that excessive water resulted in unstable emulsions [17]. Overall, research on the subject has been mostly restricted to lab-scale experiments, and no scaling-up efforts have been made regarding any of these systems. In addition, very few writers have been able to systematically address the microstructure of the emulsion and successfully identify the spatial distribution of the wax in the final system.

In this paper, the potential of waxes to form water-in-oleogel emulsions without added emulsifiers is described. First, by means of a drop shape analysis, we will analyze an array of different natural waxes to identify systems that interact more with the water phase. Then, the experiments will be narrowed to BZW and CRW, where lab-scale systems will be prepared to further elucidate the underlying mechanisms behind wax-stabilized W/O emulsions. Finally, the scaling-up potential will be assessed by crystallizing the systems in a pilot-scale scraped surface heat exchanger (SSHE).

## 2. Materials and Methods

### 2.1. Materials

Sunflower oil was received from Vandemoortele R&D (Izegem, Belgium). White beeswax (BZW), berry wax (BEW), candelilla wax (CLW), carnauba wax (CRW), and sunflower wax (SFW) were acquired from C.E. Roeper GmbH (Hamburg, Germany). Oryza sativa rice bran wax (RBW) was provided by Kalhwax GmbH & Co. KG (Trittau, Germany). In all emulsions, deionized water was used as the dispersed phase.

### 2.2. Methods

#### 2.2.1. Contact Angle Measurements

For the two-phase contact angle measurements, wax slides of 1 mm thickness were prepared. Hot wax was melted 15 °C above the melting point and poured into a tray forming a 1 mm thick lamina. Squares of 1 × 1 cm were cut to form the wax slides. A micropipette was used to dispense 10 µL of deionized water on top of the wax slides. One hour of stabilization time was allowed before measurement. Drop shape analysis was performed with a Krauss DSA 10 MK2 (KRÜSS GmbH, Hamburg, Germany) that was available at the Particle and Interfacial Technology Group research group (Ghent University). Image analysis was performed on sessile drops using a Young–Laplace fit. The resulting contact angle measurement was the average of two droplets, where each drop was measured three times.

#### 2.2.2. Critical Gelling Concentration (CGC)

For the preparation of the oleogels, wax and oil were carefully weighted and combined. The CRW systems contained 3%, 3.5%, 4.0%, 4.5%, and 5% *w*/*w* of wax, while the BZW systems were tested at 0.5%, 1.0%, 1.5%, 2.0% and 3.0% *w*/*w*. Next, the dispersions were heated to 15 °C above the wax’s melting temperature under constant magnetic stirring (500 rpm) until no solid particles were visible (CRW systems were heated to 98 °C, while BZW systems were heated to 80 °C). The oleogel was then poured into small cylindrical containers (15 mL) and immediately refrigerated (5 °C) for an hour. To identify the critical gelling concentration (CGC), samples were inverted and kept under refrigeration (5 °C) for one hour. CGC was determined as the sample with the lowest concentration and no visible mobility [20]. Two replicates were performed.

#### 2.2.3. Lab-Scale Emulsion Preparation

For the preparation of lab-scale emulsions, a wax–oil blend was prepared and heated to 15 °C above the wax melting temperature under constant stirring (CRW systems were heated to 98 °C, while BZW systems were heated to 80 °C). In a separate container, water was heated to the same temperature. Emulsions (200 g) were then prepared by mixing the melted wax–oil dispersions with the water under high shear (2 min at 11,000 rpm) using an Ultraturrax (IKA T 25 digital ULTRA-TURRAX^®^, IKA-Werke GmbH & Co. KG, Staufen, Germany). The composition information is available in Table 1. Since rapid crystallization has been reported to enhance emulsion stability [16,21], the samples were placed in 15 mL storage containers and immediately placed under deep-freezing conditions (−18 °C) for 15 min to achieve fast cooling. The samples were then stored at 5 °C until measurement (Figure S1).

#### 2.2.4. Pilot-Scale Emulsion Preparation

For the preparation of emulsions on a pilot scale, a two-step process was used, consisting of a pre-emulsification step and a dynamic crystallization step (Figure S1). For the pre-emulsification step, oil and wax were heated to 90 °C under constant magnetic stirring. Simultaneously, water was heated separately to 90 °C. When both phases reached the same temperature, a pre-emulsion was prepared by slowly incorporating the aqueous phase into the wax–oil blend using an IKA Eurostar 20 mechanical stirrer with an axial flow-pitched blade impeller (400 rpm). The pre-emulsion was kept at 98 °C for the next step. The composition information is available in Table 1. In each production, around 4 kg of sample was prepared.

For the crystallization step, a benchtop scraped surface heat exchanger (SSHE) was used (Het Stempel B.V, Zwijndrecht, The Netherlands). Two main components constitute the process. Firstly, there is a micropump unit with a speed range of 16 to 2000 rpm, a maximum pressure of 80 bar, and an operating voltage of 380 V. The second component is a micro SSHE unit with a volume of 29 mL, two sets of scraping knives on the rotor axis, a motor of 1.1 kW and a rotational speed of up to 2000 rpm. This device allows fat blend systems that mimic industrial SSHE to be produced. For further specifications, the reader is referred elsewhere [22]. Free water on the surface of the samples was evaluated utilizing a Wator qualitative test paper (MACHEREY-NAGEL GmbH & Co. KG, Düren, Germany). The operating parameters were tailored to the intrinsic conditions of each system. For the CRW experiments, 15 rpm of pump speed, 1500 rpm in the SSHE unit, and a cooling temperature of 5 °C were used. The flow rate was kept between 2.0 and 2.5 Kg/h. The inlet temperature was rigorously controlled at 98 °C. For the BZW experiments, a pump speed of 40 rpm was used, followed by 250 rpm in the SSHE unit and a cooling temperature of 5 °C. The inlet temperature was carefully monitored at 75 °C and the flow rate was kept between 5.0 and 5.3 Kg/h.

#### 2.2.5. Stability Index

A so-called stability index (SI) was used to measure the stability of the emulsions. The emulsions were placed in narrow cylindrical glass containers. The stability index, expressed as a percentage, was calculated as the ratio between the emulsion height (hE) and the total height (hT), which comprised creamed oil, sedimented water, and emulsion (Equation (1)) [23]. The results were the average value of five replicates.

SI(%) = hE/Ht·100%(1)

#### 2.2.6. Rheology

Rheological properties were determined at 5 °C using an MCR Anton Paar 302 (Anton Paar, Graz, Austria) with a Peltier system for temperature control. A 25 mm sandblasted parallel plate geometry was used (PP25S). Samples were transferred from the storage container into the center of the geometry with the help of a spatula, shearing as little as possible in order to avoid excessive structural damage. The gap was set to 1 mm, and the excess sample was trimmed. A recovering time of 5 min was employed before each measurement. Strain sweeps were conducted from 0.01% to 100% at a constant angular frequency of 10 rad/s. Storage (G′), loss (G″) and complex (G*) moduli were recorded as a function of shear strain. The limit of the linear viscoelastic region (LVR) was found when G′ or G″ had a 5% deviation from their initial value. The strain at the LVR limit is referred to as the “yield strain” or linearity limit (γ_L_). The crossover point or flow point (γ_F_) refers to the moment at which G′ = G″ (after that, G″ > G′, and thus, the system is in a fluid state). Each experiment was performed in triplicate.

#### 2.2.7. PLM

Polarized light microscopies (PLM) were taken using a Leica DM2500 microscope (Wetzlar, Germany) equipped with a Leica MC170 HD color camera. A small amount of sample was placed between a glass slip and a coverslip, and placed under the microscope. All images were taken using a 20× magnification lens. PLM was used to validate whether there were any microscopical changes throughout the storage time. At least 5 different images were acquired each time.

#### 2.2.8. Confocal Laser Scanning Microscopy Visualization (CLSM)

Nile Red (Sigma Aldrich, St. Louis, MO, USA) was used as a fluorophore to stain the continuous fat phase. A stock solution of Nile Red in acetone of 0.01% *w/v* was prepared. An aliquot of 25 µL of the stock solution was applied on a glass slide cover, spread evenly and let to evaporate under ambient conditions. After the complete evaporation, a sample of W/O emulsions was carefully placed on the glass slide and softly pressed to ensure complete contact with the evaporated Nile Red solution. Next, the sample was stored at 5 °C in darkness for 1 h to allow the diffusion of the fluorophore into the continuous fat phase. The samples were visualized using a Nikon A1R confocal microscope (Nikon Instruments, Apris, France), which was mounted on a Nikon Ti body and equipped with a 60× Plan Apo oil immersion objective (numerical aperture 1.4). The excitation laser for Nile Red was 560, and the detection was performed in the range of 570–738 nm. To make sure that the images were representative of the sample, at least 20 different droplets were evaluated per sample.

#### 2.2.9. Cryo-SEM

For the visualization via cryogenic electron scanning microscopy (cryo-SEM), the emulsions were placed on aluminum stubs and vitrified in a liquid nitrogen slush (−210 °C). The stub with the sample was then transferred under vacuum conditions into a PP30100T cryo-transfer system for the SEM preparation stage (Quorum Technologies Ltd., East Sussex, UK), which was conditioned at −140 °C. In the preparation stage, the sample was fractured, sublimated (−70 °C for 90 min) and sputter-coated with platinum using argon gas. The visualization was performed in a Jeol JSM-7100F TTLS LV TFEG-SEM (Jeol Europe BV, Zaventem, Belgium), under high-vacuum conditions (1 × 10^−6^ mbar), at −140 °C and at an accelerated voltage of 3 keV. To make sure the images were representative of the sample, at least 50 different water droplets were evaluated per sample.

#### 2.2.10. Statistical Analysis and Data Processing

Python was used to perform all statistical analyses. In all cases, a level of significance of 0.05 was used. The Shapiro–Wilk test was performed to review normality, while homoscedasticity was evaluated using Levene’s test. When assumptions were met, a one-way ANOVA was used to compare the mean values, and a post hoc analysis was performed utilizing Tukey’s HSD. Otherwise, a Wilcoxon signed-rank test was used, followed by a post hoc Dunn test.

## 3. Results and Discussion

The results will be presented in four different sections:

1. Preliminary studies were aimed at reducing the number of experiments. Water–wax interactions were quantified by means of contact angle measurements. Two waxes were selected, and CGC was used to determine the relevant wax concentrations.
2. Lab-scale experiments were used to assess the viability of the emulsions prepared using an Ultraturrax on a lab scale.
3. Pilot-scale experiments aimed to assess the scaling-up potential of the W/O emulsions. Here, the most promising system was prepared using a pilot-scale SSHE and further evaluated.
4. Microstructural analyses were performed in order to understand the fat crystal spatial distribution and the underlying interactions between the wax oleogels and water.

### 3.1. Preliminary Experiments

Table 2 presents the results of the two-phase contact angle measurement sorted by magnitude. CLW, BEW and RBW reported higher contact angles, reflecting a more hydrophobic nature. In contrast, BZW, CRW and SFW, displayed lower angles, suggesting a higher degree of interaction with the water phase. Based on these results, BZW and CRW were selected as potential candidates for stabilizing W/O emulsions without added emulsifiers. No further experiments were conducted using CLW, BEW, RBW or SFW. For BZW, the obtained CGC was 1.5%, while for CRW, the CGC was 5% (Figure S2).

### 3.2. Lab-Scale Experiments

Based on the preliminary experiments, eleven different emulsions were prepared. Three 20% W/O emulsions were prepared per type of wax using concentrations that were equivalent to 50% of the CGC, 100% of the CGC and 150% of the CGC. Hence, the BZW emulsions contained 0.75%, 1.5% and 2.25% wax in the continuous phase, while the CRW emulsions were formulated at 2.5%, 5% and 7.5% *w*/*w*. The motivation behind this selection was to obtain the following:

- One emulsion with wax concentrations well below the CGC,
- One emulsion with wax concentrations well above the CGC,
- One emulsion with the exact amount required for gelling the oil phase.

To compare the performance of both waxes at the same concentration, an additional 20% W/O emulsion with 5% BZW in the continuous phase was prepared. Furthermore, to elucidate the role of water in the emulsion’s structure, 30% and 40% of W/O emulsions were included in the study with the same wax concentration (%*w*/*w*) for CRW and BZW. Results were then compared with the respective oleogel. A compilation of all lab-scale systems is available in Table 3.

The physical stability of the emulsions over one month of storage was assessed utilizing the SI (Table 3). All of the emulsions prepared with CRW were stable regardless of the concentrations of water and wax used. In contrast, BZW emulsions with wax concentrations of 0.75% and 1.5% failed the stability test, displaying phase separation (SI < 5%). BZW emulsions prepared at higher concentrations of wax (2.25% and 5%) could hold physical integrity during the storage time, even at higher volumes of water. In conclusion, when present in a sufficient concentration, both waxes could stabilize lab-scale W/O emulsions over one month of storage. The results support the findings of Binks and Rocher (2009) and Szumała andLuty (2009) for CRW and those of Gao et al. (2021) for BZW emulsions [16,17,19].

**Figure 1:**
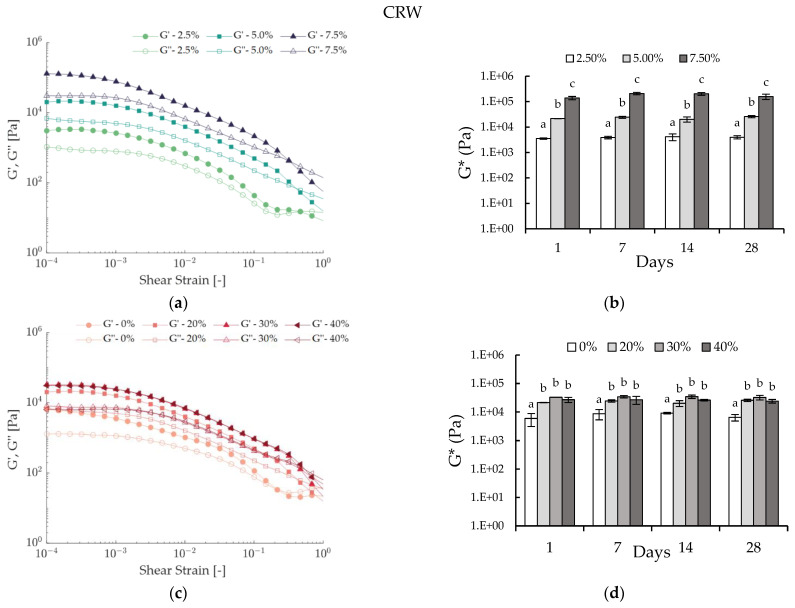
Rheological results of lab-scale CRW emulsions. (**a**) Strain sweeps illustrating the effect of wax concentration on 20% W/O emulsions one day after preparation; (**b**) Effect of wax concentration on the complex modulus (G*) of 20% water CRW emulsions during storage time. Means with different letters are significantly different (*p* < 0.05); (**c**) Strain sweep test illustrating the effect of water concentration on 5% CRW emulsions one day after preparation; (**d**) Effect of water concentration on the complex modulus (G*) of 5% CRW emulsions during storage time. Means with different letters are significantly different (*p* < 0.05).

Figure 1 presents the rheological results of lab-scale CRW emulsions. Increasing amounts of wax led to higher G′ and G″ values, thus creating more rigid systems. Figure 1a,b confirm this finding, as the addition of wax significantly increased G* (*p* < 0.05). A number of studies reported equivalent results for oleogel systems, as an increasing amount of wax led to higher structured systems [24,25,26,27]. The same conclusion was then extrapolated to water-in-oleogel emulsions [17]. Nonetheless, there were no significant differences in the γ_L_ of the CRW emulsions with different concentrations (see Figure S3), suggesting that an increase in the amount of wax had a minimal impact on the yield point of the emulsions.

**Figure 2:**
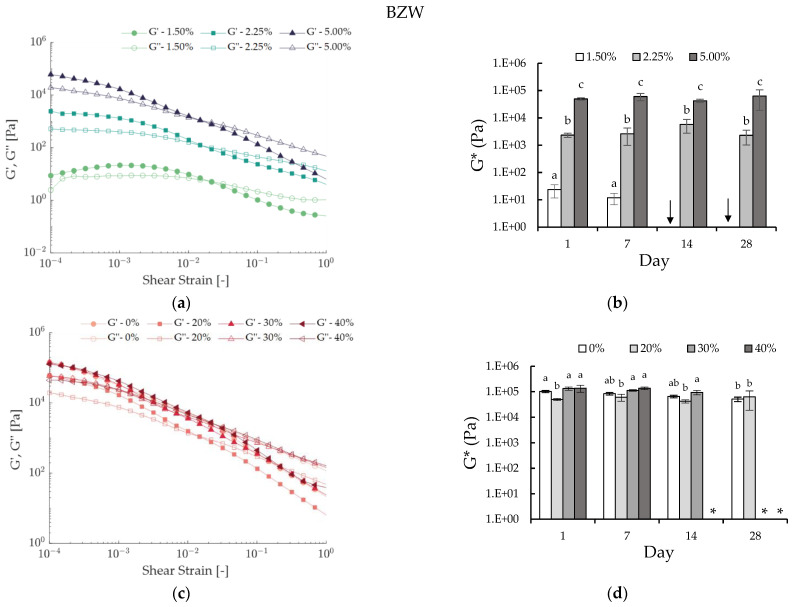
Rheological results of lab-scale BZW emulsions. (**a**) Strain sweep test illustrating the effect of wax concentration on 20% water BZW emulsions one day after preparation.; (**b**) Effect of wax concentration on the complex modulus (G*) of 20% water BZW emulsions during storage time. Means with different letters are significantly different (*p* < 0.05); (**c**) Strain sweep test illustrating the effect of water concentration on 5% BZW emulsions one day after preparation; (**d**) Effect of water concentration on the complex modulus (G*) of 5% BZW emulsions during storage time. Means with different letters are significantly different (*p* < 0.05). The asterisk and arrows denote emulsions that could not be measured due to physical instability (arrow) (Table 3) or shear sensitivity (*) (Figure S4).

Similar to the case of CRW, an increasing wax concentration considerably increased the G′ and G″ of all the BZW emulsions (Figure 2a,b). Hence, this supports the hypothesis that the wax concentration is a critical factor in the emulsion rigidity and G*. However, when comparing the strain sweeps of the CRW emulsions (Figure 1a) with the BZW emulsions (Figure 2a), the results suggest that the BZW produced less stable systems. The BZW emulsions reported consistently lower yield strains than CRW (see Figure S3). Interestingly, an increasing wax concentration did not seem to have a significant impact on the yield strain, thus suggesting that the shear sensitivity was dictated by the nature of the wax (see Figure S3).

In the case of the CRW emulsions, there was no significant increase or decrease in the G* associated with storage time (*p* > 0.05), and therefore the prepared CRW emulsions were regarded as stable within the tested concentration range and time (Figure 1b,d). Similarly, BZW emulsions prepared at higher concentrations (≥2.25% of wax) were stable over one month of storage. However, for the BZW systems, physical stability was not always achieved. Emulsions prepared at a CGC of 100% were not measured due to phase separation (see arrows in Figure 2b). In some cases, although there was no visible phase separation (SI = 100%), the sample would collapse upon compression when placed in the rheometer (Figure S4), and, therefore, the results were no longer deemed reliable (see asterisks in Figure 2d). This phenomenon can probably be attributed to compression-induced coalescence, as droplet mobility increases when the emulsion undertakes shear [28].

There are several factors that were found to be influencing the rheological properties of W/O emulsions [29,30]. Rousseau (2020) propounds that three main contributors are dictate the rheological properties: the “matrix” (continuous phase), the filler (dispersed aqueous phase) and the processing/usage parameters [30]. Intrinsically, if the G* of the matrix (oleogel) increases with an increasing wax concentration, the G* of the composite (W/O emulsion) will also increase (as described in Figure 1b). Moreover, the dispersed droplets can act as “active” or “inactive” fillers. When attractive interactions exist between the filler and the matrix, the composite strength will increase, and the water droplets will act as active fillers (thus, the G* of the composite will be higher than the G* of the matrix). This can occur when interfacial crystallization takes place, and has been previously identified in specific wax W/O emulsions [14,30,31]. In contrast, inactive fillers might not have an important effect on the rheological modulus; hence, the G* of the composite will be equal to or less than the G* of the matrix [15,30].

When comparing Figure 1c and Figure 2c, we can infer that both systems responded differently to increasing fractions of the dispersed phase. In the case of BZW, the addition of water led to weaker or equal rigidity (Figure 2d), while for CRW, the overall G* increased (*p* < 0.05) (Figure 1d). This result suggests that in the BZW emulsions, the water phase acted as an inactive filler, as the overall modulus of the composite was determined by the dispersed phase, thus implying a weaker interaction between water droplets and the wax crystal matrix. The opposite occurred in the CRW emulsions, thus suggesting that the dispersed water phase acted as an active filler in that system.

### 3.3. Pilot-Scale Experiments

**Figure 3:**
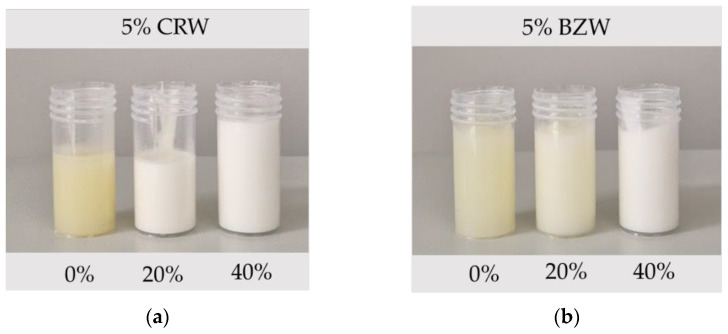
Picture of pilot-scale oleogels and emulsions on day 430 of storage. (**a**) 5% CRW emulsions with 0%, 20% and 40% of water, respectively (from left to right). (**b**) 5% BZW emulsions with 0%, 20% and 40% of water (from left to right). Images show how prepared emulsions have minimal phase separation.

**Figure 4:**
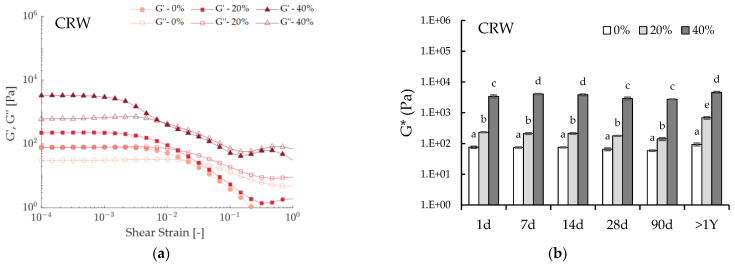

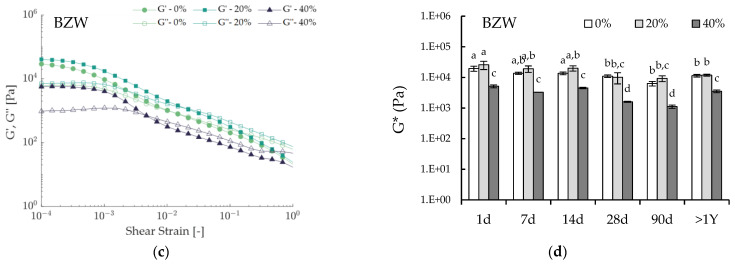
Rheological results of emulsions prepared with a SSHE. (**a**) Strain sweep test illustrating the effect of water concentration on 5% CRW emulsions one day after preparation; (**b**) Effect of water concentration on the complex modulus (G*) of 5% CRW systems during storage time. Means with different letters are significantly different (*p* < 0.05); (**c**) Strain sweep test illustrating the effect of water concentration on 5% BZW emulsions one day after preparation; (**d**) Effect of water concentration on the complex modulus (G*) of 5% BZW emulsions during storage time. Means with different letters are significantly different (*p* < 0.05).

Pilot-scale experiments are relevant as they provide industrially relevant processing conditions and help determine scale-up potential. In addition, due to the inherently different crystallization protocol (simultaneous rapid cooling and shearing), samples prepared on a pilot scale tend to be more homogeneous than those prepared statically [7]. Six systems were crystallized on a pilot-scale SSHE: 5% wax emulsions were prepared at 0% (oleogel), 20% and 40% water per type of wax. All systems were regarded as stable (SI = 100%), as no visible phase separation was encountered throughout the one year of storage time (Figure 3), and the rheological parameters remained unchanged (Figure 4b,d).

In accordance with the lab-scale results, BZW emulsions reacted differently when water was added. Increasing amounts of water in the CRW emulsion led to significantly higher moduli (Figure 4), thus further supporting the idea that water droplets act as active fillers in that system. On the contrary, the increase in the dispersed phase in the BZW emulsions led to decreasing or equivalent rigidities compared to the oleogel, thus corroborating the idea that water acts as an inactive filler. In other words, while the water droplets and the oleogel in the CRW emulsions contribute synergistically to promoting network building, in the BZW emulsions, the network structure is mostly determined by the oleogel. Water droplets can be regarded as “structural defects” that either have no effect or hinder the gelling capacity of the BZW oleogel. In all rheological experiments on pilot scale samples, compression led to no water loss before analysis.

**Figure 5:**
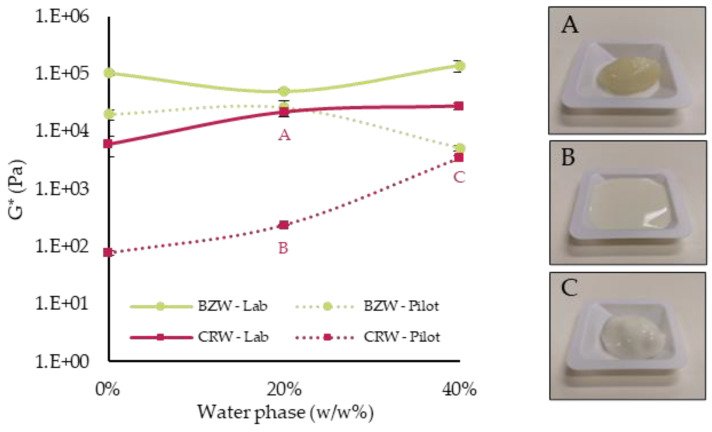
Complex Moduli (G*) as a function of the water phase concentration (%*w*/*w*) on day 1. All emulsions were prepared at 5% wax. In the images: (**A**) 5% CRW, 20% water, lab scale; (**B**) 5% CRW, 20% water, pilot scale; and (**C**) 5% CRW, 40% water, pilot scale. Images (**A**,**B**) compare two emulsions with the same composition, one prepared on a lab-scale (**A**) and one prepared on a pilot scale (**B**). Images (**B**,**C**) compare two different emulsions prepared on a pilot scale with different water concentrations, 20% (**B**) and 40% (**C**). Error bars represent one standard deviation.

Systems produced on a pilot scale displayed substantially lower moduli values than those prepared on a lab scale (see Figure 5, comparison between dotted and full lines). This is regarded as an important finding in terms of scaling up applications, as crystallization under shear had an enormous impact on the macroscopic characteristics of the wax oleogels and oleogel emulsions (see images A and B in Figure 5). A similar conclusion can be drawn when comparing the strain sweeps, as the production method also impacted the structural breakdown. For instance, the CRW oleogel had a yield stress of 16.32 ± 2.08 Pa when prepared statically and a yield stress of 0.43 ± 0.04 when prepared on a pilot scale. Similarly, for the CRW emulsion prepared with 20% water, the yield strain was 23.27 ± 4.04 Pa when prepared in the lab, while the same system prepared in the pilot plant had a yield strain of 0.78 + 0.04 Pa. Overall, when produced in the benchtop SSHE, the systems displayed a weaker network, as lower shear strains were observed at the linearity limit.

### 3.4. Microstructural Studies

**Figure 6:**
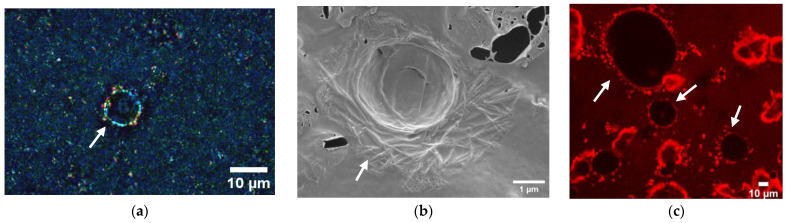

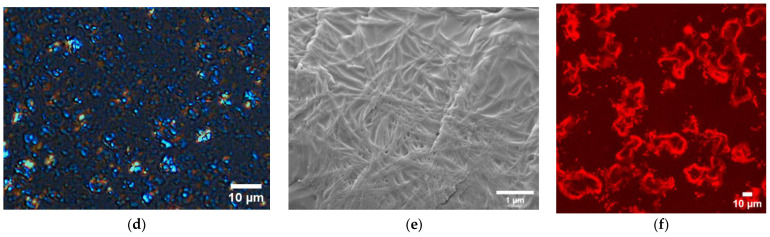
Microstructure of CRW. (**a**) PLM and (**b**) Cryo-SEM images of a 5% CRW emulsion (40% water) produced on a pilot scale; (**c**) CSLM images of a 5% CRW emulsion (40% water) prepared on a lab scale. Arrows indicate the location of the CRW crystals. (**d**) PLM and (**e**) Cryo-SEM images of a 5% CRW oleogel prepared on the SSHE; (**f**) CSLM image of a 5% CRW oleogel prepared on a lab scale.

**Figure 7:**
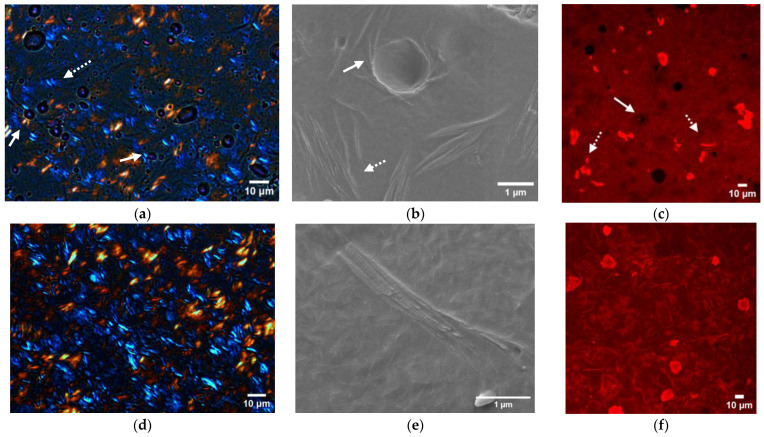
Microstructure of BZW. (**a**) PLM and (**b**) Cryo-SEM images of a 5% BZW emulsion (20% water) produced on a pilot scale; (**c**) CSLM images of a 5% BZW emulsion (40% water) produced on a lab scale. Full arrows indicate the location of BZW crystals in contact with water droplets. Dashed arrows indicate BZW crystals that are independent from the water phase. (**d**) PLM and (**e**) Cryo-SEM images of a 5% BZW oleogel produced on a pilot scale; (**f**) CSLM image of a 5% CRW oleogel crystallized on a lab scale.

The idea behind this section is to elucidate how, although both emulsions achieved physical stability, their rheological behavior is different. As such, the performance of the W/O emulsions stabilized with fat crystals is strongly linked to the fat crystal spatial distribution. Ghosh and Rousseau (2011) described three possible arrangements in which fat crystals can stabilize W/O emulsions. First, when the lipid has surface-active properties, it will be absorbed onto the water–oil interface and behave as a Pickering particle. This allows the formation of a physical barrier, preventing droplet coalescence and emulsion destabilization (Pickering emulsion). On the other hand, surface inactive crystals will not interact with the water droplets. Instead, when they are in sufficiently large concentrations, they will form a three-dimensional network upon cooling. This will then increase viscosity and enclose the droplets, preventing droplet diffusion and hindering destabilization. This arrangement is known as “network stabilization”. A third approach consists of the synergy of the first two mechanisms, in which Pickering stabilization and network stabilization occur simultaneously [32]. In order to elucidate which type of arrangement occurs in each system, the microstructure was carefully revised by utilizing three different techniques: PLM, Cryo-SEM and CLSM. Figure 6 and Figure 7 present a compiled view of the microscopies for the CRW and BZW emulsions, respectively, and their corresponding oleogel.

Figure 6d–f illustrate the oleogel system. From these images, we can identify how the wax is recognized in each technique. From Figure 6a–c, we can infer that CRW crystals are often placed in the vicinity of the water droplet (see arrows). The finding is systematically repeated in each visualization technique, as evidenced by a higher birefringence in the PLM, the presence of a clear network in the Cryo-SEM and small particles in the CLSM, all surrounding water droplets. The presence of wax solid particles in the water interface suggests that CRW crystals act as a Pickering stabilizer in the absence of added emulsifiers. This, combined with the gelling properties of CRW, support the hypothesis that there is a synergic effect between the water and the CRW oleogel (Pickering and network stabilization), thus potentially explaining the emulsion’s high resistance to coalescence.

Similarly, Figure 7d–f displays the BZW oleogel. In the PLM (Figure 7a), the BZW crystals are visualized as needle-like shapes. This is in accordance with previous studies conducted by Öğütcü et al. [33], and Doan et al. [20]. In spite of this, research that offers contradictory findings has emerged. The Cryo-SEM analysis of RBW, SFW and CLW, which are also visualized as needle-like crystals under the PLM, revealed a platelet-like morphology [34,35]. Altogether, we cannot conclude that the BZW crystals had needle-like structures, as this result might come from microscopy artifacts.

Turning now to the emulsion system (Figure 7a–c), the location of the BZW crystals seems to be independent of the position of the water droplets. As such, we can find wax crystals both in the proximity of the water interface (filled arrows), as well as far from them (dashed arrows). Although the BZW crystals do not seem to be repelled by the water phase, there are no indications that the BZW acts as a Pickering particle. Especially revealing are the CLSM images, where the overall microstructure does not seem to change in the presence of water. Hence, it could be hypothesized that the oleogel network stabilizes the BZW emulsions and that water acts as an inactive filler. This, therefore, explains why, when compressed, the emulsion loses all structural integrity and collapses (as described in the rheological section, see Figure S4).

**Figure 8:**
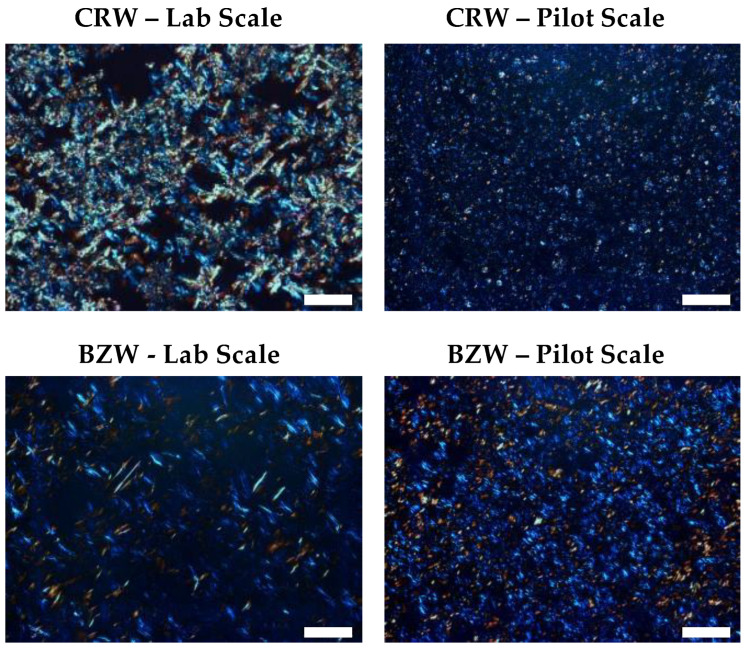
PLM of pilot-scale and lab-scale-produced oleogels. Scalebar = 50 μm.

Finally, based on the microscopies, we could infer that the systems produced on a pilot scale had distinctly smaller crystallite sizes than those prepared on a lab scale (Figure 8). Due to the differences in the processing conditions, the intricate crystal network is changed, resulting in a lower moduli and visibly different macroscopic behaviors (Figure 4). Although similar conclusions were obtained by Alvarez-Mitre et al. when crystallizing CLW at shear rates above 600 s^−1^ [36], these findings cannot be extrapolated to all shearing–cooling conditions, concentrations and wax systems [24,37]. We hypothesize that the loss in structure is linked to the prolonged shear strain applied after nucleation, causing crystal erosion and limiting the development of permanent junction zones [5].

## 4. Conclusions

This work demonstrated that BZW and CRW could stabilize W/O emulsions in the absence of added surfactants. First, contact angle experiments helped quantify an array of different wax–water interactions. BZW, CRW and SFW displayed lower angles, of which BZW (CGC = 1.5%) and CRW (CGC = 5.0%) were selected for further experimentation. Next, lab-scale emulsions were prepared at three different concentrations (below the CGC, above the CGC and at the CGC). The physical stability of the emulsions over one month of storage was assessed visually and rheologically. CRW systems were stable regardless of the wax or water concentration tested (always above 2.5%, equivalent to 50% of the CGC), while BZW emulsions needed concentrations of wax above 150% of the CGC (>2.25%) to hold physical integrity. Overall, increasing amounts of wax led to higher G′ and G″ values, as the wax concentration was the main factor affecting emulsion rigidity. In the case of BZW, the addition of water led to weaker or equal rigidity, suggesting that water acted as an inactive filler in the system. On the contrary, for CRW, the overall G* increased with water addition (*p* > 0.05), suggesting that the dispersed phase acted as an active filler. Similar trends were found in the pilot-scale experiments. Emulsions produced on a pilot scale showed no visible phase separation over the one year of storage; however, they displayed substantially lower moduli values (G*) compared to those prepared on a lab scale. Imaging techniques, such as Cryo-SEM, CLSM and PLM, were utilized to elucidate the underlying structure–function relationships. CRW emulsions seemed to be stabilized by a synergy between Pickering and network stabilization. In contrast, for the BZW emulsions, network stabilization occurred when the BZW was present in sufficiently large concentrations. It is believed that these results will assist to improve understanding of wax-stabilized W/O emulsions. The future successful implementation of these findings will help to attain healthier fat products with a high added value.

## Figures and Tables

**Table 1:** Composition of lab-scale and pilot-scale experiments.

Preparation Method	Wax	Water Concentration (*w*/*w*%)	Wax Concentration (*w*/*w*% Oil Phase)
Lab Scale	CRW	0	5.00
		20	2.50
			5.00
			7.50
		30	5.00
		40	5.00
	BZW	0	5.00
		20	0.75
			1.50
			2.25
			5.00
		30	5.00
		40	5.00
Pilot Scale	CRW	0	5.00
		20	
		40	
	BZW	0	
		20	
		40	

Abbreviations: CRW: Carnauba wax; BZW: beeswax.

**Table 2:** Contact angle measurement and critical gelling concentration (CGC).

Wax	Contact Angle Measurement (°)	CGC (%*w*/*w*)
CLW	107.08 ± 1.87	Not measured
BEW	105.65 ± 2.56	Not measured
RBW	103.93 ± 1.19	Not measured
SFW	88.05 ± 1.88	Not measured
CRW	87.87 ± 1.15	5.0
BZW	83.57 ± 3.38	1.5

Abbreviations: CLW: candelilla wax; BEW: berry wax, RBW: rice bran wax, SFW: sunflower wax, CRW: carnauba wax; BZW: beeswax, CGC: critical gelling concentration.

**Table 3:** Stability index (SI) of lab-scale emulsions. Emulsions that failed the stability test are indicated with an asterisk (*). Emulsions with a 100% stability index showed no phase separation during storage time.

Wax	Water Concentration (*w*/*w*%)	Wax Concentration (*w*/*w*% Oil Phase)	Time (Days)			
			1	7	14	30
CRW	20%	2.50 (=50% CGC)	100.0%	100.0%	100.0%	100.0%
		5.00 (=100% CGC)	100.0%	100.0%	100.0%	100.0%
		7.50 (=150% CGC)	100.0%	100.0%	100.0%	100.0%
	30%	5.00 (=100% CGC)	100.0%	100.0%	100.0%	100.0%
	40%	5.00 (=100% CGC)	100.0%	100.0%	100.0%	100.0%
BZW	20%	0.75 (=50% CGC)	100.0%	99.3% *	96.2% *	95.9% *
		1.50 (=100% CGC)	100.0%	100.0%	98.2% *	96.7% *
		2.25 (=150% CGC)	100.0%	100.0%	100.0%	100.0%
		5.00 (=333% CGC)	100.0%	100.0%	100.0%	100.0%
	30%	5.00 (=333% CGC)	100.0%	100.0%	100.0%	100.0%
	40%	5.00 (=333% CGC)	100.0%	100.0%	100.0%	100.0%

Abbreviations: CRW: carnauba wax; BZW: beeswax, CGC: critical gelling concentration.

## Data Availability

The data presented in this study are openly available in Zenodo at 10.5281/zenodo.7503685.

## Supplementary Materials

The following supporting information can be downloaded at: https://www.mdpi.com/article/10.3390/foods12091850/s1, Figure S1: Diagram of Preparation techniques; Figure S2: Critical gelling concentration (CGC) experiments; Figure S3. Yield strain of CRW and BZW W/O emulsions prepared at 50% of the CGC, 100% of the CGC, 150% of the CGC and 333% of the CGC on a lab scale (20% of water); Figure S4. Compression-induced coalescence while during sample preparation for rheological measurements.
