# Supplementary material for: Carnauba Wax and Beeswax as Structuring Agents for Water-in-Oleogel Emulsions without Added Emulsifiers
Source: Foods. 2023 Apr 29;12(9):1850. doi: 10.3390/foods12091850 (PMC10178762; doi:10.3390/foods12091850)
Supplement: Supplementary file 1 [file foods-12-01850-s001.zip › foods-2274218-supplementary.pdf]

Supplementary Material

Carnauba Wax and Beeswax as Structuring Agents for Water-in-Oleogel Emulsions without Added Emulsifiers

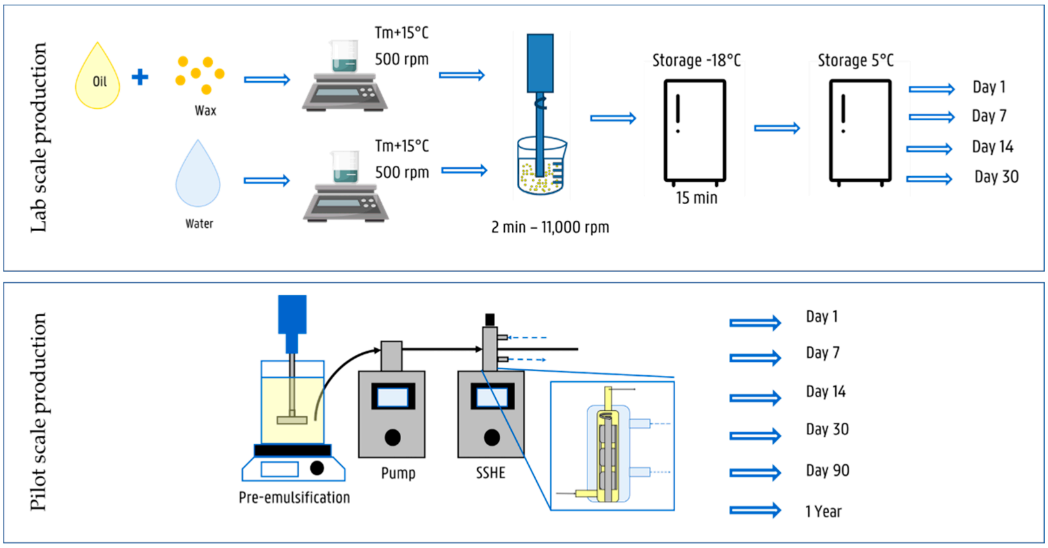

Abbreviations: Tm = Melting Temperature, SSHE = Scraped surface heat exchanger, rpm= revolutions per minute.

Figure S1. Diagram of Preparation techniques

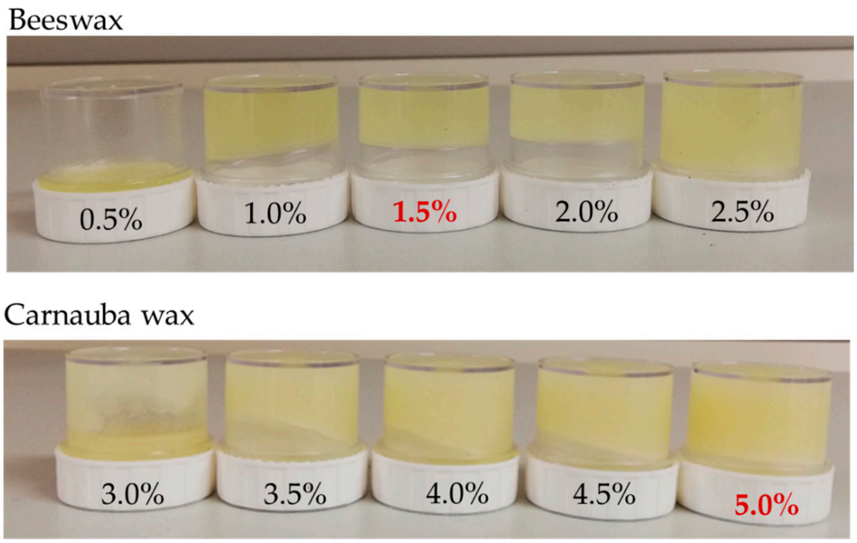

Figure S2. Critical gelling concentration (CGC) experiments. The red text indicates the CGC of each wax.

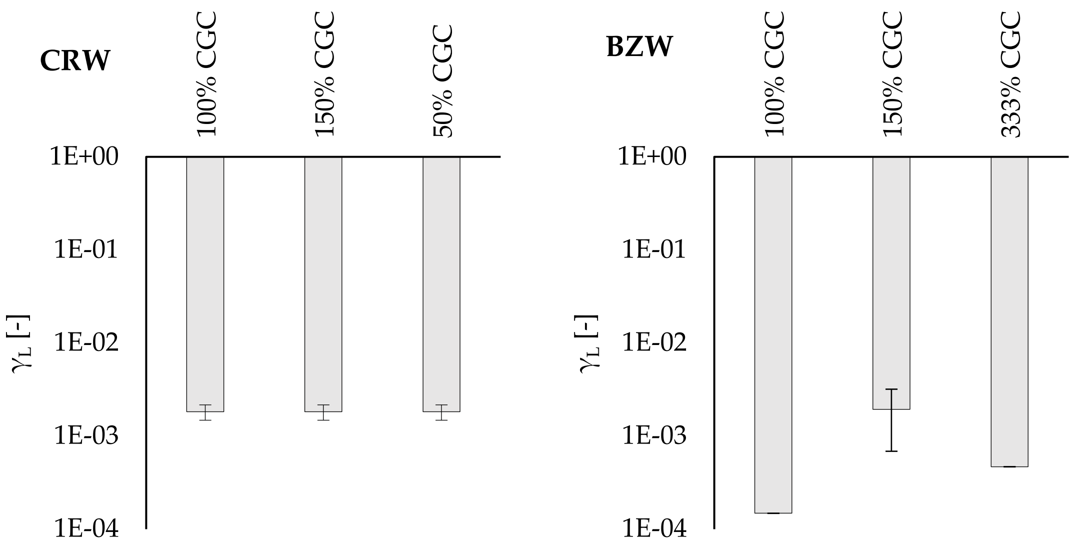

Figure S3. Yield strain of CRW and BZW W/O emulsions prepared at 50% of the CGC, 100% of the CGC, 150% of the CGC and 333% of the CGC on a lab-scale (20% of water). Error bars represent one standard deviation. There are no significant differences between the three concentration levels ($p>0.05$).

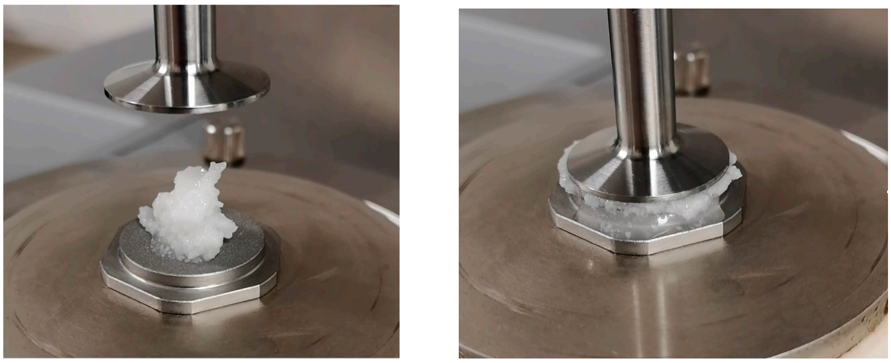

Figure S4. Compression-induced coalescence while during sample preparation for rheological measurements.
